# Expression of miR-1-3p, miR-16-5p and miR-122-5p as Possible Risk Factors of Secondary Cardiovascular Events

**DOI:** 10.3390/biomedicines9081055

**Published:** 2021-08-20

**Authors:** Rafał Badacz, Paweł Kleczyński, Jacek Legutko, Krzysztof Żmudka, Jacek Gacoń, Tadeusz Przewłocki, Anna Kabłak-Ziembicka

**Affiliations:** 1 Department of Interventional Cardiology, Institute of Cardiology, Jagiellonian University Medical College, John Paul II Hospital, 31-202 Krakow, Poland; rbadacz@gmail.com (R.B.); kleczu@interia.pl (P.K.); jacek.legutko@uj.edu.pl (J.L.); zmudka@icloud.com (K.Ż.); jgacon@o2.pl (J.G.); tadeuszprzewlocki@op.pl (T.P.); 2 Department of Invasive Cardiology, E. Szczeklik’s Hospital, 33-100 Tarnow, Poland; 3 Department of Cardiac and Vascular Diseases, Institute of Cardiology, Jagiellonian University Medical College, John Paul II Hospital, 31-202 Krakow, Poland; 4 Noninvasive Cardiovascular Laboratory, John Paul II Hospital, 31-202 Krakow, Poland

**Keywords:** acute ischemic event, biomarkers, cardiovascular events, cardiovascular death, carotid artery lesions, coronary artery disease, prognostic circulating miRs, recurrent myocardial infarction and ischemic stroke

## Abstract

Ischemic event in one arterial territory increases the risk of a subsequent ischemic event. Circulating microRNAs (miRs) emerge as a potential clinical tool to assess risk of subsequent atherothrombotic events such as cardiovascular death (CVD), myocardial infarction (MI) and ischemic stroke (IS). In this prospective study, we searched for athero-specific miRs related to cardiovascular event risk in patients with symptomatic coronary, carotid lesion, or both territories involvements. The choice of particular miRs was based on database research (Pub-Med, Bethesda, MD, USA) taking into consideration the relationship with development of atherosclerosis and potential prognostic value. Levels of circulating miRs (miR-1-3p, miR-16-5p, miR-34a-5p, mir-122-5p, miR-124-3p, miR-133a-3p, miR-133b, miR-134-5p, miR-208b-3p, miR-375 and miR-499-5p) were compared in 142 patients with an acute ischemic event resulting from carotid and/or coronary artery stenosis, who underwent revascularization for symptomatic lesion. A 6-year prospective evaluation of CVD/MI/IS risk was performed. Patients with two-territory as compared to single-territory involvement differed in levels of miR-1-3p (*p* = 0.016), miR-16-5p (*p* < 0.001), miR-34a-5p (*p* = 0.018), miR-122-5p (*p* = 0.007), miR-124-3p (*p* < 0.001) and miR-499-5p (*p* < 0.001). During follow-up, 62 (43.7%) episodes of CVD/MI/IS occurred. In multivariate Cox analysis, miR-122-5p (HR = 1.0006, 95%CI = 1.0001–1.0011) and peripheral artery disease (PAD) (HR = 2.16, 95%CI = 1.26–3.70) were associated with CVD/MI/IS risk; miR-1-3p (HR = 2.73, 95%CI = 1.22–6.12) and PAD (HR = 3.47, 95%CI = 1.88–6.41) with CVD; miR-122-5p (HR = 1.0001, 95%CI = 1.000–1.0002) and creatinine level (HR = 1.02, 95%CI = 1.01–1.04) with IS, and miR-16-5p (HR = 1.0004, 95%CI = 1.0001–1.0008) with MI. Expression of miR-1-3p, miR-16-5p and miR-122-5p during incident ischemia may be possible risk factors of secondary cardiovascular event(s).

## 1. Introduction

Atherosclerosis leading to athero-thrombotic events constitutes a major cause of fatal and non-fatal hospital stays as well as pre-hospital sudden deaths [1]. Furthermore, survivors of myocardial infarction (MI) and ischemic stroke (IS) are at higher risk for recurrence of adverse events [2,3,4].

It is estimated that approximately 5.3 to 6.6% and 29% have recurrent MI at 1 and 5 years respectively, while the risk of recurrent stroke is about 10% at 1 year and 25% at 5 years [2,5,6].

Recurrent MI and IS are associated with greater mortality risk compared to a primary event [2,3,4,5]. Immediate evaluation of patients with MI or IS and treatment initiation is crucial for survival. As IS treatment depends on the underlying cause, in case of athero-thrombosis of the carotid artery, carotid revascularization for 70–99% of symptomatic carotid stenosis is associated with 48% risk reduction for IS recurrence at 1 year [4]. In line, revascularization of coronary artery culprit lesion is vital for the patient’s hemodynamic stabilization and survival [7].

Ischemic event in one arterial territory increases the risk of subsequent ischemic events in another vascular bed [8,9]. Therefore, secondary prevention of recurrence is a high priority. Although a vast work-up of biochemical, imaging and clinical predictors of outcomes were established, in a substantial number of patients prognosis after incident MI and IS remain unpredictable [10,11].

One of the contemporary directions is the identification of specific genetic biomarkers. Particularly, circulating microRNAs (miRs), a family of small non-coding RNAs, deserve further exploration, as they play an enormous regulatory role in controlling posttranscriptional expression of their target genes. Their pathological down- or up-regulation is responsible for carcinogenesis, atherothrombosis, neurological disorders and many other diseases [12,13,14,15].

Thus, circulating miRs emerged as potentially important clinical tools to assess the risk of a subsequent atherothrombotic event, e.g., cardiovascular death (CVD), MI and IS, following the primary event. The down- or up-expression of miRs has an impact on atherosclerosis and athero-thrombosis processes, through induction or suppression of vascular inflammation, apoptosis, endothelial dysfunction, smooth muscle cells proliferation, angiogenesis, plaque progression and rupture [14,15].

We hypothesize that the release of specific miRs as a consequence of the ischemic event in one territory may lead to transformation and, in consequence, to the destabilization of atherosclerotic plaques in the same or other arterial territories.

Therefore, in the present study, we searched for athero-specific miRs related to risk of forthcoming cardiovascular events in patients with either symptomatic coronary or carotid lesion, or both territories, involvements in a 6-year follow-up period.

## 2. Materials and Methods

### 2.1. Study Population

In this prospective study, 142 patients with diagnosis of MI and IS admitted between January 2013 and January 2014 were evaluated. In all patients, revascularization of culprit lesion was performed, followed by 6 years of follow-up for outcomes.

Group I comprised 47 patients with primary symptomatic carotid atherothrombotic disease causing 70–99% stenosis, who underwent carotid artery stenting or carotid endarterectomy according to guidelines [16].

Group II comprised 40 patients admitted with coronary plaque rupture, who underwent urgent coronary angiography with subsequent percutaneous coronary intervention according to guidelines [17].

Group III consisted of 55 patients with an athero-thrombotic event within the carotid artery with concomitant coronary artery steno-occlusive plaques that were revascularized in both arterial territories.

Inclusion criteria included biochemical, ECG and clinical findings relevant to myocardial infarction accompanied by at least one culprit lesion in the coronary artery on coronary angiography. This was followed by a primary percutaneous coronary angioplasty with stent implantation and/or appropriate neurologic symptoms associated with carotid stenosis exceeding 70% lumen reduction in the territory of cerebral ischemia with relevant brain imaging findings.

Exclusion criteria included acute heart failure or congestive heart failure in NYHA classes III and IV, non-atherosclerotic myocardial infarction, no link between carotid stenosis and neurological deficits on examination or lesions on brain CT tomography, any active cancer disease, systemic inflammatory conditions, such as arthritis, and known or suspected bacterial or viral infections.

**Figure 1:**
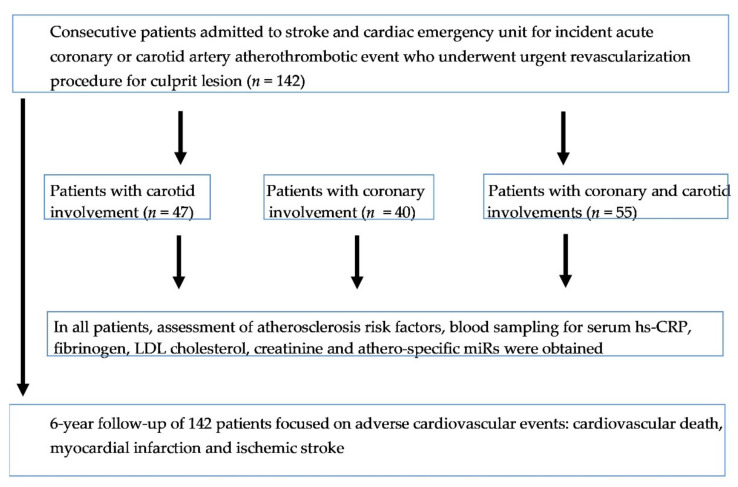
Study flowchart.

A study flowchart is presented in Figure 1.

The distribution of classic risk factors (diabetes, hyperlipidemia, arterial hypertension, smoking current or previous, renal kidney disease and peripheral occluded arterial disease) was recorded. Definitions of the above were adopted from the scientific statements of the European Society of Cardiology [18,19,20].

All patients obtained peri- and post-procedural optimal medical treatment according to recommendations of respective societies.

The study was performed in accordance with the Declaration of Helsinki. The protocol was reviewed and approved by a local ethics committee.

### 2.2. Biochemical Tests and miRs Extraction

All patients had fasting blood samples obtained on patient admission to the department, prior to any intervention and immediately after the signed informed consent was obtained from the patients.

The standard blood tests included assessment of high-sensitivity C-reactive protein (hs-CRP), fibrinogen, creatinine and low-density-lipoprotein (LDL) cholesterol levels.

Peripheral blood from serum samples for profiling miRs was collected on patient admission before heparin treatment. Samples were allowed to coagulate for 30 min, centrifuged and sera were frozen at −80 °C until miRNA analysis.

Extraction of miRs was performed by means of the miRNeasy Serum/Plasma Kit (cat. No. 217184, Qiagen, Hilden, Germany) with the beginning lysis by Trizol LS Reagent (cat. No. 10296-028, Invitrogen, Waltham, MA, USA). The RNA yield and concentrations were determined by capillary electrophoresis on the Agilent Bioanalyser 2100 with the Eukaryote Total RNA Pico Chip (Agilent Technologies, Inc, Santa Clara, CA, USA). An average of 60 ± 31.9 pg/μL total RNA from 300 μL of serum was recovered.

### 2.3. Profiling of miRs

The following circulating miRs were analyzed in each case: miR-1-3p, miR-34a-5p, miR-122-5p, miR-124-3p, miR-133a-3p, miR-133b, miR-134-5p, miR-208b-3p, miR-375, miR-499-5p and a reference endogenous miR-16-5p. The primers sequence and catalog numbers of miRs are given in Supplementary Material (Table S1).

Our choice for selection of particular miRs was based on database research (PubMed, Bethesda, MD, USA). Analyzed miRs were taken into consideration based on the data regarding their potential relationship with development of atherosclerosis (usually based on experience in patients with coronary and peripheral artery disease) and their potential prognostic value. Additionally, we included miRs that were discriminating biomarkers for coronary artery vessel occlusion in patients with acute coronary syndrome [21].

At the time of the study, Exiqon LNA primers were used to quantify 10 mature miRs using the ViiA 7 real-time PCR system equipped with a 384-well reaction plate (Life Technologies, Carlsbad, CA, USA). RNA was converted to cDNA using the Universal cDNA Synthesis Kit (cat. No. EQ-203300, Exiqon, Vedbæk, Denmark). Before synthesis, RNAs were spiked with a synthetic miRNA that served as a control for the cDNA synthesis reaction. Real-time PCR was performed in triplicate with SYBR Green master mix Universal RT (cat. No. EQ-203400, Exiqon, Vedbæk, Denmark) using standard conditions.

Data were processed by the delta-Ct method, using a global normalization approach as implemented in the open source DataAssist software (Life Technologies, Carlsbad, CA, USA). The fold changes (RQ) were calculated, and statistically significant variations between group samples were filtered by the calculation of adjusted *p*-values using the Benjamini–Hochberg false discovery rate.

### 2.4. Outcome Data, Follow-Up and Adverse Cardiovascular Events

The incidences of CVD, MI and IS as well as composite end-point (CVD/MI/IS) were recorded prospectively during a follow-up period of 6 years. Adverse events were defined as fatal or non-fatal IS, fatal or non-fatal MI, or CVD (i.e., any sudden or unexpected death unless proven as non-cardiovascular on autopsy). MI was diagnosed according to criteria of the European Society of Cardiology. Diagnosis of IS was to be given by a neurologist to ensure reliability.

Final visits were done through telephone contact with a patient or appointed family member. One patient was lost to follow-up; however, the data on patient vital status were obtained from the national health registry.

### 2.5. Statistical Analysis

Continuous variables are presented as mean ± one standard deviation (SD). Categorical variables are expressed as frequencies and percentages. Means of analyzed parameters across groups were tested with the analysis of variance (ANOVA) test, and frequencies were compared by the chi-square test for independence.

The potential independent prognostic markers of cardiovascular events during the follow-up period were established from the clinical, biochemical and miRs variables with a Cox proportional hazard univariate analysis, and in case of a trend toward difference (*p* < 0.1), they were entered into a multivariate Cox proportional hazard analysis model. The results of the multivariate logistic regression analysis were expressed as hazard ratio (HR) and 95% confidence interval (95%CI).

Statistical analyses were performed with Statistica 13.0 software. Statistical significance was assumed at *p*-value < 0.05.

## 3. Results

### 3.1. Patient Characteristics

The baseline characteristics of the patients are summarized in Table 1.

Group II patients were younger, as compared to patients with carotid involvement (*p* < 0.001) and two-territory involvements (*p* < 0.001), and they had lower prevalence of hypertension (*p* = 0.026 and *p* = 0.005, respectively). There were no significant differences across groups with regard to distribution of gender, smoking, diabetes, hyperlipidemia, concomitant peripheral artery disease and renovascular disease. However, LDL cholesterol level was higher in Group II patients compared to Group I (*p* < 0.001) and Group III (*p* < 0.001). There was a higher concentration of hs-CRP (*p* = 0.017) in coronary vs. carotid involvement. Patients with two-territory involvements had higher creatinine levels compared to coronary involvement patients (*p* = 0.023).

**Figure 2:**
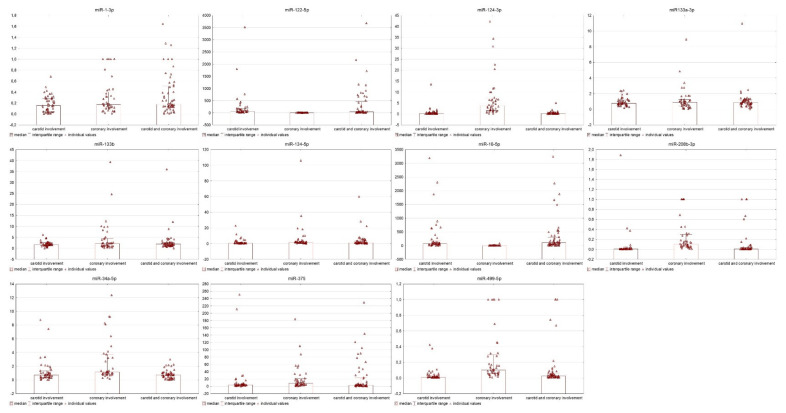
Detected miRs for every study group (carotid involvement, coronary involvement and carotid and coronary involvements) shown as dot plot bar-graphs with individual dots of each individual patient to find the tendency of miRs expressions in each group. Each dot represents one sample.

Patients with two-territory involvements as compared to single-territory involvement differed in levels of miR-1-3p (*p* = 0.016), miR-16-5p (*p* < 0.001), miR-34a-5p (*p* = 0.018), miR-122-5p (*p* = 0.007), miR-124-3p (*p* < 0.001) and miR-499-5p (*p* < 0.001) (Figure 2).

### 3.2. Clinical, Biochemical and miRs Parameters and the Outcomes

Prospective evaluation of CVD, recurrent MI and IS incidence was accomplished for all patients. During the 6-year follow-up period, CVD/MI/IS occurred in 62 (43.6%) subjects, including 44 (31%) CVDs, 20 (13.8%) non-fatal MI and 12 (8.3%) non-fatal IS.

Age, diabetes, renal and peripheral artery disease, carotid and coronary involvements, levels of hs-CRP and serum creatinine, miR-1-3p, miR-16-5p and miR-122-5p showed association with adverse cardiovascular events in the univariable Cox proportional hazard analysis (Table 2).

The multivariate Cox proportional hazard analysis revealed that miR-122-5p (HR 1.001, 95%CI 1.0001–1.0011, *p* = 0.017) and peripheral artery disease (HR 2.16, 95% CI 1.26–3.70, *p* = 0.004) were associated with CVD/MI/IS incidence (Table 2).

Risk of CVD was associated with levels of miR-1-3p (HR 2.73, 95%CI 1.22–6.12, *p* = 0.014) and peripheral artery disease (HR 3.47, 95%CI 1.88–6.41, *p* < 0.001), while MI was associated with miR-16-5p (HR 1.0004, 95%CI 1.0001–1.0008, *p* = 0.011).

IS risk was related to miR-122-5p (HR 1.0001, 95%CI 1.000–1.0002, *p* = 0.024) and serum creatinine level (HR 1.02, 95%CI 1.01–1.04, *p* < 0.001).

## 4. Discussion

The novelty of our study is that expression of miR-1-3p, miR-16-5p and miR-122-5p, analyzed in patients during an acute ischemic athero-thrombotic event, may have prognostic value for secondary ischemic events at long-term follow-up. Our findings support the hypothesis that genetic posttranscriptional expression of their target genes play an important regulatory role in atherosclerotic cardiovascular disease [22]. Once miRs release is initiated, they exert long-term influences on progression and destabilization of atherosclerotic plaques that were clinically irrelevant at the moment of acute ischemia.

The other major finding in the present study is that, despite the same athero-thrombotic etiology resulting in an ischemic event, the levels of circulating miRs significantly differed between the studied groups.

Two-territory involvements (symptomatic carotid and coronary athero-thrombotic lesions) were associated with higher expression of miR-16 and miR-122, as compared to ischemia induced by coronary arteries. Similarly, carotid artery-related cerebral ischemia produced much more miR-16 and miR-122 than coronary arteries. In contrast, patients with acute coronary ischemia had higher expression of miR-34a and miR-124. Patients with symptoms from the carotid artery were characterized with lower expression of miR-1. This finding might be potentially attributed to the different mechanisms of coronary and carotid ischemia [22,23,24].

With regard to atherosclerotic processes and adverse cardiac events, few miRs have been identified as modulators of different processes directly involved in atherosclerotic plaque instability and rupture, and this is particularly unbalanced for high-risk patients [13]. In the recent study, miR-24 (HR 3.842, *p* = 0.011) strongly predicted major cardiac adverse events at 2 years of follow-up [13].

In the present study, we demonstrated high expression of circulating miR-16-5p in patients with carotid territory ischemia that was associated with increased risk of future MI. Potential explanation of this phenomenon is that high expression of miR-16-5p in patients with atherosclerosis-related cerebral ischemia may initiate hydrogen peroxide (H_2_O_2_)-induced myocardial injury, triggering coronary artery oxidative stress [25,26]. However, the interplay between down- and up-regulation of miR-16 seems much more complex.

Although miR-16 is typically highly expressed in cardiomyocytes, miR-16-5p levels were low in patients with acute coronary ischemia [27]. This may be a positive finding, as downregulation of miR-16 protects cardiomyoblast H9C2 cells against the oxidative stress induced by H_2_O_2_ [25]. In line, Liu et al. demonstrated, in rats with acute coronary ischemia caused by coronary artery ligation, a protective role of miR-16 against acute MI by reversing beta2-adrenergic receptor down-regulation [28]. Similarly, high expression of miR-16 in ApoE^−/−^ mice fed a high-fat diet showed an anti-inflammatory effect of miR-16 on interleukine-6, and it was negatively associated with the severity of coronary artery disease [29].

Thus, although miR-16 is generally thought to have a protective role against atherosclerotic processes, by inhibiting the inflammatory pathways [29], especially by reducing the IL-6 levels, while promoting secretion of IL-10 and TGF-beta, we observed associations between high miR-16 expression and future MI. On the other hand, in the study by Tian et al. [30], miR-16 levels were associated with hyperacute cerebral infarction incidence as compared to healthy controls, which means that the role of miR-16 in the atherosclerotic process, including oxidative stress and plaque transformation, leading to coronary and cerebral ischemia remains unestablished and requires further assessment.

Researchers found the involvement of sirtuin 1 (SIRT1) and plaque miR-33 on the pro-inflammatory and pro-coagulable state of the coronary thrombus in hyperglycemic patients with ST-elevation acute coronary syndrome, as well as the expression of miR-92a as a vital contributor to the activation of cardiac fibroblasts in post-MI patients [31,32]. These findings are clinically relevant because miR-33 expression directly at the level of atherosclerotic plaque acts as modulator of inflammatory markers and molecular pathways implied in the pathogenesis of coronary thrombus in patients with acute MI [31].

In our present study, we observed associations between miR-122-5p release and future episodes of CVD/MI/IS and IS incidence. In the recent review by Ali Sheikh et al., concerning the therapeutic value of miRs in coronary artery disease, miR-122-5p is considered, among other liver-derived miRs, to regulate lipoprotein metabolism. Therefore, the altered expression of miR-122 may be a co-factor of dyslipidemia, leading to adverse cardiac and cerebral ischemic events [33]. In fact, in patients with coronary ischemia, miR-122 expression was low, consistent with low incidence of IS during the follow-up in this subset of patients, while it was high in the carotid ischemia group and associated with high IS recurrence.

In our study miR-1 appeared as the prognostic factor of composite end-point (CVD/MI/IS) as well as CVD during follow-up. Although the prognostic effect of miR-1 on MI during follow-up did not reach statistical significance in the multivariate analysis, its prognostic role in the cardiovascular outcome seems important. miR-1 affects coronary circulation mainly by regulating the endothelial function and angiogenesis progression. In the review by Navickas et al., miR-1 was considered a potential biomarker of acute coronary ischemia [34]. Moreover, the miR-1 expression level was correlated with worse MI outcome in the studied populations.

Of note, miRs could be over-expressed in high-risk patients also at the level of organs not directly involved in atherosclerotic processes, such as the peripheral adipose tissue [35,36]. In this setting, the adipose tissue could work as an active gland in the synthesis and relapse of miRs that negatively influence cardiac performance and atherosclerotic processes.

Consistently, the expected clinical benefit in patients with an acute ischemic event would be the introduction of specific medical treatment, which either would modify the expression of miRs resulting in better risk factor control or reduction in atherosclerotic burden, or specific miRs inhibitors to modify target miRs expression [13,35]. In line, in obese pre-diabetics, metformin significantly reduces inflammation/oxidative stress, and miR-195 and miR-27 were associated with reduction in left ventricle mass and intima-media thickness [36].

One obvious issue to discuss is recurrence of adverse cardiovascular events despite obtained revascularization for culprit lesions. Although our study population included patients with ischemia-related symptoms of carotid and coronary artery occlusive disease that were relieved by index carotid and/or coronary revascularization, the incidence of CVD/MI/IS during the 6-year follow-up period was high, accounting for 43.7%.

The problem of recurrent cardiovascular events despite intervention is well known [2,3]. In recent reviews of literature, ischemia recurrence occurred in 10.0% vs. 21.2% of patients with complete vs. culprit coronary lesion revascularization at 12 months [2].

Survivors of MI or IS are at high risk for recurrence, other cardiovascular events or death. An analysis of the FOURIER trial demonstrated that high-risk patients with a history of coronary artery disease and maximally tolerated statin therapy are at substantial risk for the recurrence of multiple cardiovascular events [37]. Among survivors of first MI between 2004 and 2010 in England, recurrence rates were 5.6% for men and 7.2% for women at 1 year, and 13.9% and 16.2%, respectively, at 7 years [38]. Other studies of IS recurrence have reported cumulative recurrence rates of 8.0 to 12.6% at 1 year, 10.8 to 12.1% at 2 years, and 16.6% at 5 years [38,39,40,41,42]. Among patients surviving an IS, the incidence of all-cause death was 24.5% at 1 year and 41.3% at 4 years [39].

Bye and colleagues postulated a panel of five miRs that revealed an enhanced risk prediction of suffering a fatal MI in putatively healthy individuals in a 10-year observation period [43]. With this panel, 78% of the observed cases were correctly classified. These authors suggested using the miRs expression levels of miR-106a-5p, miR-424-5p, let-7 g-5p, miR-144-3p and miR-660-5p that improved the predictive power of the Framingham scale even further, i.e., increased the area under the curve from 0.72 to 0.91 [43]. Conversely, we have previously published a tool for discrimination between healthy subjects and patients with coronary artery disease, which may be used for identification of subjects at risk for exercise-induced cardiac events. Using miRs resting expression levels of miR-150-5p as well as the modulated expression levels after a maximal ergometry of miR-101-3p, miR-141-3p and miR-200b-3p, 70% of the cases were correctly classified as patients with coronary artery disease. Including the performance parameters of maximal oxygen uptake as well as maximum exercise capacity corrected for the bodyweight the correct classification of coronary artery disease cases was raised to 92.5% [44].

Mechanisms of acute ischemic events are complex, depending on many co-factors and inter-players; however, the regulatory role of miRs during one ischemic event should be taken into consideration as a potential risk factor for future ischemic events.

One obvious limitation of the present study is lack of multiple assessments that would allow for evaluation of longitudinal changes in circulating miRs. Repeated evaluation of miRNAs in patients with acute ischemic events could provide further information on the miRNA activity and stability, which should be undertaken in future studies.

## Figures and Tables

**Table 1:** Study group characteristics.

	Group I Carotid Involvement *N* = 47	Group II Coronary Involvement *N* = 40	Group III Carotid and Coronary Involvements *N* = 55	ANOVA *p*-Value
**Demographic data**				
Age, (years)	68.8 ± 9.7	57.3 ± 10.1	68.7 ± 9.2	<0.001 *; 0.996 **; <0.001 ***
Male, *n* (%)	27 (57%)	27 (68%)	37 (67%)	0.597 *; 0.561 **; 0.999 ***
Smoking habit, *n* (%)	27 (57%)	26 (65%)	36 (65%)	0.751 *; 0.686 **; 0.998 ***
Hypertension, *n* (%)	44 (94%)	31 (78%)	53 (96%)	0.026 *; 0.882 **; 0.005 ***
Diabetes, *n* (%)	14 (30%)	19 (48%)	23 (42%)	0.210 *; 0.429 **; 0.841 ***
Hypercholesterolemia, *n* (%)	42 (89%)	34 (85%)	45 (82%)	0.837 *; 0.537 **; ***0.903
Renal artery stenosis, *n* (%)	13 (28%)	6 (15%)	9 (16%)	0.629 *; 0.496 **; ***0.105
Peripheral artery disease, *n* (%)	13 (28%)	5 (13%)	15 (27%)	0.215 *; 0.998 **; ***0.210
**Laboratory data**				
Serum creatinine, (µmol/L)	86.3 ± 31.0	76.8 ± 15.5	91.4 ± 28.9	0.222 *; 0.599 **; 0.023 ***
C-reactive protein, (g/L)	4.07 ± 8.02	10.32 ± 16.5	5.83 ± 6.38	0.017 *; 0.681 **; 0.104 ***
LDL cholesterol, (mmol/L)	2.62 ± 1.04	3.83 ± 1.32	2.71 ± 0.93	<0.001 *; 0.923 **; <0.001 ***
Fibrinogen, (g/L)	3.88 ± 1.34	4.02 ± 1.71	3.78 ± 1.37	0.909 *; 0.937 **; 0.729 ***
**miR expression**				
miR-1-3p, A.U.	0.18 ± 0.15	0.31 ± 0.31	0.35 ± 0.37	0.147 *; 0.016 **; 0.756 ***
miR-122-5p, A.U.	244.1 ± 592	1.94 ± 3.01	338.7 ± 664.8	0.095 *; 0.664 **; 0.007 ***
miR-124-3p, A.U.	0.77 ± 2.06	7.36 ± 9.64	0.44 ± 0.74	<0.001 *; 0.950 **; <0.001 ***
miR-133a-3p, A.U.	0.96 ± 0.55	1.23 ± 1.58	1.48 ± 3.10	0.832 *; 0.453 **; 0.839 ***
miR-133b, A.U.	1.95 ± 1.15	4.45 ± 7.38	2.95 ± 4.97	0.065 *; 0.598 **; 0.342 ***
miR-134-5p, A.U.	9.75 ± 50.9	6.37 ± 17.4	3.79 ± 9.20	0.869 *; 0.606 **; 0.914 ***
miR-16-5p, A.U.	155 ± 225	3.25 ± 11.7	149 ± 175	<0.001 *; 0.986 **; <0.001 ***
miR-208b-3p, A.U.	0.07 ± 0.29	0.24 ± 0.32	0.64 ± 2.78	0.887 *; 0.251 **; 0.539 ***
miR-34a-5p, A.U.	1.95 ± 5.16	4.85 ± 9.97	0.77 ± 0.65	0.076 *; 0.604 **; 0.004 ***
miR-375, A.U.	38.6 ± 112.7	22.15 ± 35.5	24.6 ± 35.1	0.995 *; 0.593 **; 0.546 ***
miR-499-5p, A.U.	0.38 ± 0.08	0.24 ± 0.32	0.11 ± 0.25	<0.001 *; 0.253 **; 0.018 ***
**CVD/MI/IS incidence, *n* (%) #**	18 (38.3%)	19 (47.5%)	25 (45.5%)	0.719 *; 0.755 **; 0.992 ***
CVD	15	11	18	0.606 *; 0.938 **; 0.544 ***
MI	3	11	10	0.008 *; 0.075 **; 0.301 ***
IS	6	2	8	0.200 *; 0.799 **; 0.127 ***

Data are presented as mean ± SD (standard deviation) or number (%). * is for statistical analysis of carotid involvement versus coronary involvement; ** is for difference of carotid involvement versus two-territory carotid and coronary involvements; *** is for difference of coronary involvement versus two-territory carotid and coronary involvements. #—some patients had multiple cardiovascular events, e.g., ischemic stroke and myocardial infarction; CVD/MI/IS, cardiovascular death/myocardial infarction/ischemic stroke; LDL, low-density lipoprotein; A.U., arbitrary units.

**Table 2:** Univariate and multivariate Cox proportional hazard analyses for predictors of cardiovascular events during follow-up.

Cardiovascular Adverse Events	Univariate Cox Analysis		Multivariate Cox Analysis	
	HR (95%CI)	*p*-Value	HR (95%CI)	*p*-Value
**CVD/MI/IS**				
Peripheral artery disease	2.07 (1.22–3.52)	0.007	2.16 (1.26–3.70)	0.004
miR-122-5p	1.0005 (1.0003–1.001)	0.039	1.0006 (1.0001–1.0011)	0.017
miR-16-5p	1.0003 (1.0001–1.0006)	0.010		NS
**CVD**				
Age	1.03 (1.01–1.06)	0.026		NS
Peripheral artery disease	3.19 (1.73–5.86)	<0.001	3.47 (1.88–6.41)	<0.001
Carotid and coronary involvement	1.96 (1.06–3.65)	0.032		NS
miR-1-3p	2.41 (1.05–5.52)	0.036	2.73 (1.22–6.12)	0.014
miR-16-5p	1.0003 (1.000–1.0007)	0.012		NS
**MI**				
Diabetes	2.11 (0.91–4.87)	0.081		NS
miR-1-3p	2.36 (0.78–717)	0.100		NS
miR-16-5p	1.0004 (1.0001–1.0008)	0.011	1.0004 (1.0001–1.0008)	0.011
**IS**				
Age	1.04 (0.99–1.09)	0.085		NS
Renal artery stenosis	3.48 (1.29–9.35)	0.013		NS
Creatinine level	1.02 (1.01–1.03)	0.001	1.02 (1.01–1.03)	<0.001
miR-122-5p	1.000 (0.9999–1.0001)	0.070	1.0001 (1.000–1.0002)	0.024

CVD, cardiovascular death; MI, myocardial infarction; IS, ischemic stroke; hs-CRP, high-sensitivity C-reactive protein; NS, not significant.

## Data Availability

The data presented in this study are available on request from the corresponding author. The data are not publicly available due to privacy.

## Supplementary Materials

The following are available online at https://www.mdpi.com/article/10.3390/biomedicines9081055/s1, Table S1: miR primer sequence details.
