# Supplementary material for: Expression of miR-1-3p, miR-16-5p and miR-122-5p as Possible Risk Factors of Secondary Cardiovascular Events
Source: Biomedicines. 2021 Aug 20;9(8):1055. doi: 10.3390/biomedicines9081055 (PMC8391895; doi:10.3390/biomedicines9081055)
Supplement: Supplementary file 1 [file biomedicines-09-01055-s001.zip › biomedicines-1259825-supplementary.pdf]

Supplementary Table S1. miR primer sequence details.

miR name	microRNA target sequence	Sequence Reference	Corresponding LNA™ microRNA PCR primer set (cat. No)
hsa-miR-1-3p	UGGAAUGUAAAGAAGUAUGUAU	MIMAT0000416	EQ-204344
hsa-miR-122-5p	UGGAGUGUGACAAUGGUGUUUG	MIMAT0000421	EQ-205664
hsa-miR-124-3p	UAAGGCACGCGGUGAAUGCC	MIMAT0000422	EQ-204319
hsa-miR-133a-3p	UUUGGUCCCCUUCAACCAGCUG	MIMAT0000427	EQ-204788
hsa-miR-133b	UUUGGUCCCCUUCAACCAGCUA	MIMAT0000770	EQ-204162
hsa-miR-134-5p	UGUGACUGGUUGACCAGAGGGG	MIMAT0000447	EQ-205896
hsa-miR-208b-3p	AUAAGACGAACAAAAGGUUUGU	MIMAT0004960	EQ-204636
hsa-miR-34a-5p	UGGCAGUGUCUUAGCUGGUUGU	MIMAT0000255	EQ-204486
hsa-miR-375	UUUGUUCGUUCGGCUCGCGUGA	MIMAT0000728	EQ-204362
hsa-miR-499a-5p	UUAAGACUUGCAGUGAUGUUU	MIMAT0002870	EQ-205935
